# Supplementary material for: Density-wave fronts on the brink of wet granular condensation
Source: Sci Rep. 2017 Jun 15;7:3613. doi: 10.1038/s41598-017-03844-0 (PMC5472573; doi:10.1038/s41598-017-03844-0)
Supplement: Supplementary file 1 — Supplementary Informatioin [file 41598_2017_3844_MOESM1_ESM.pdf]

Supplementary Information for

Density-wave fronts on the brink of wet granular condensation

Andreas Zippelius and Kai Huang
Experimentalphysik V, Universität Bayreuth, 95440 Bayreuth, Germany

correspondence to: kai.huang@uni-bayreuth.de

Supplementary Video S1

A typical density-wave front pattern with five coherently propagating fronts. It corresponds to the snapshot shown in Fig.3(a).

Supplementary Video S2

A close-view video showing the mobility of individual particles in the vicinity of a propagating front. It is captured with a frame rate of 500 Hz and exposure time 2 ms. The black dots correspond to tracer particles. The first frame of this video corresponds to Fig.4(a). The movie is 50 times slower than real time.
